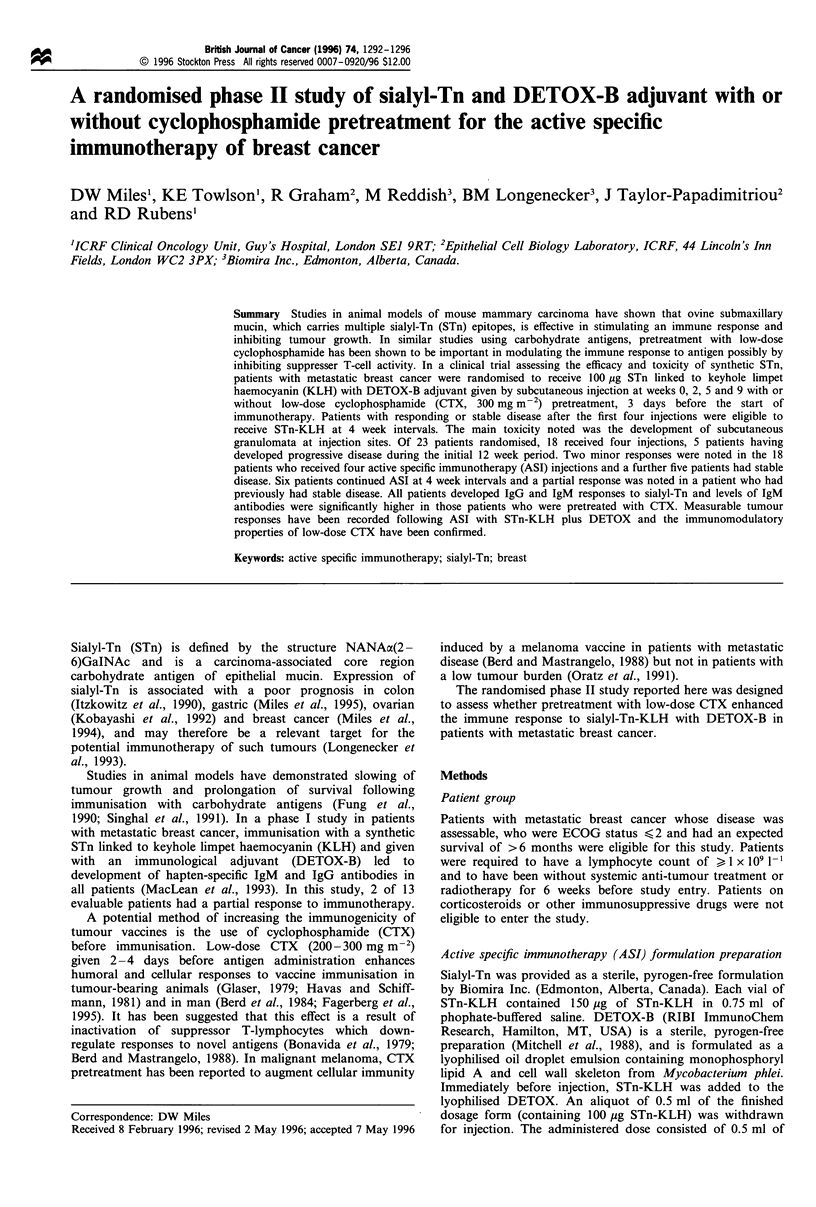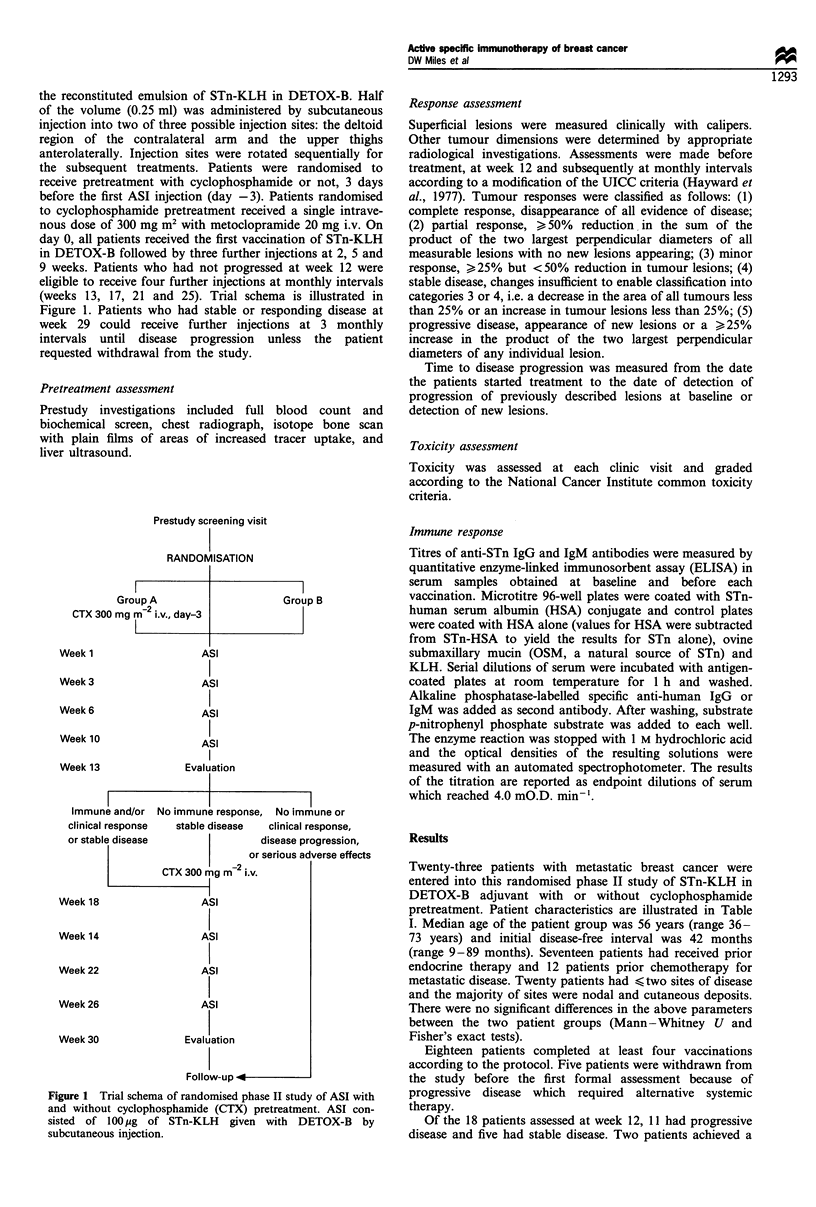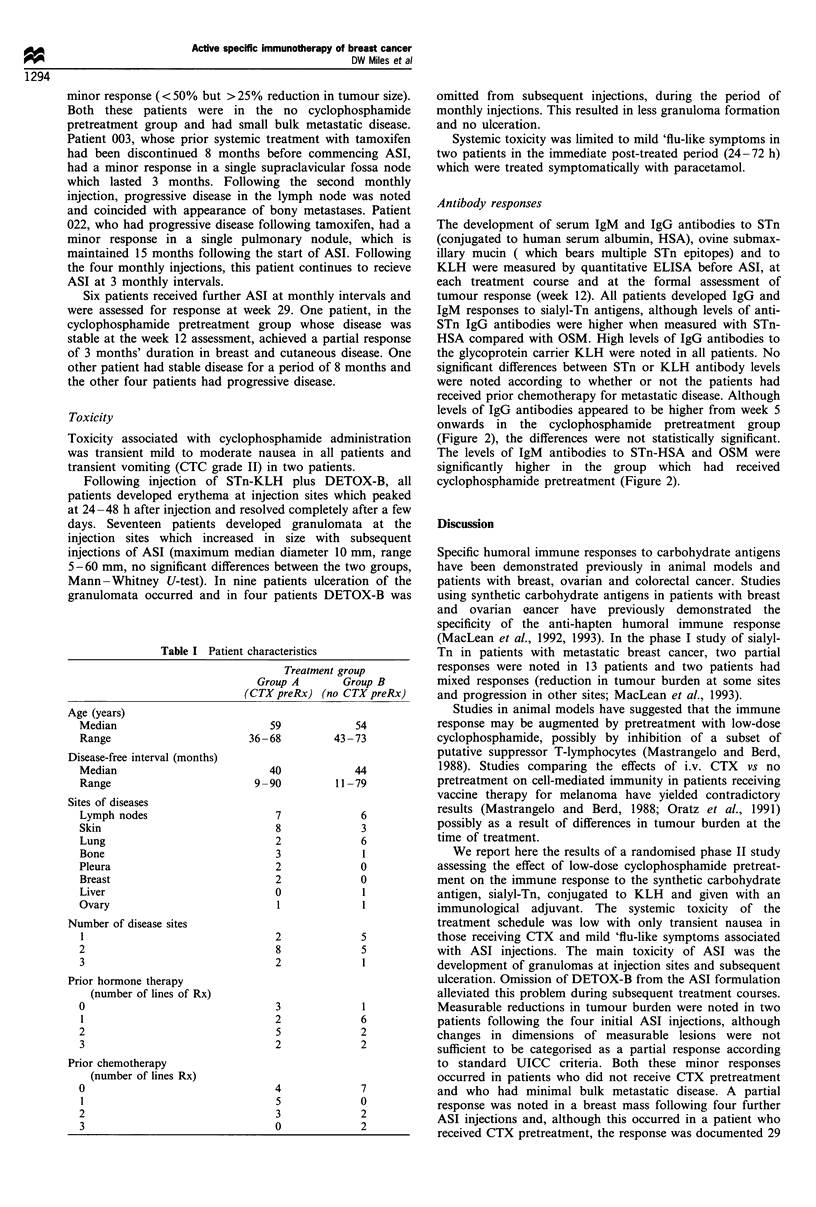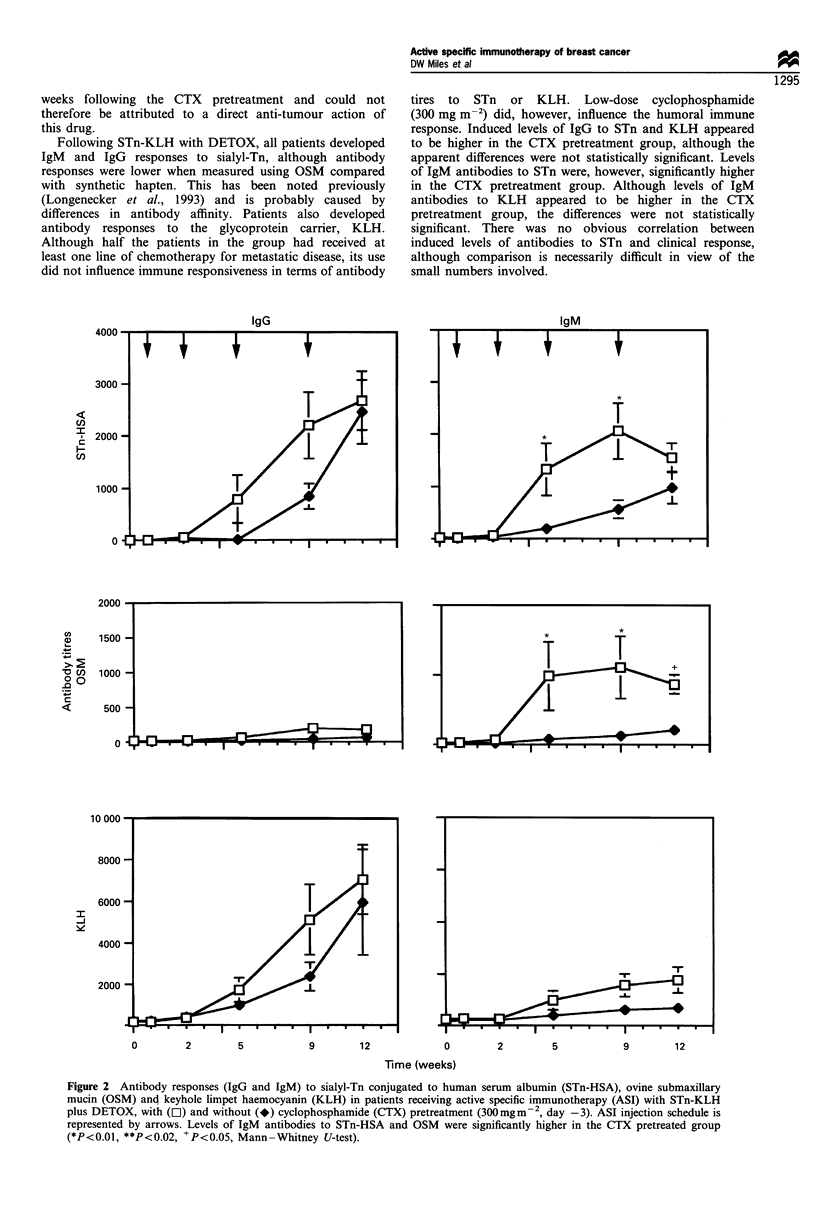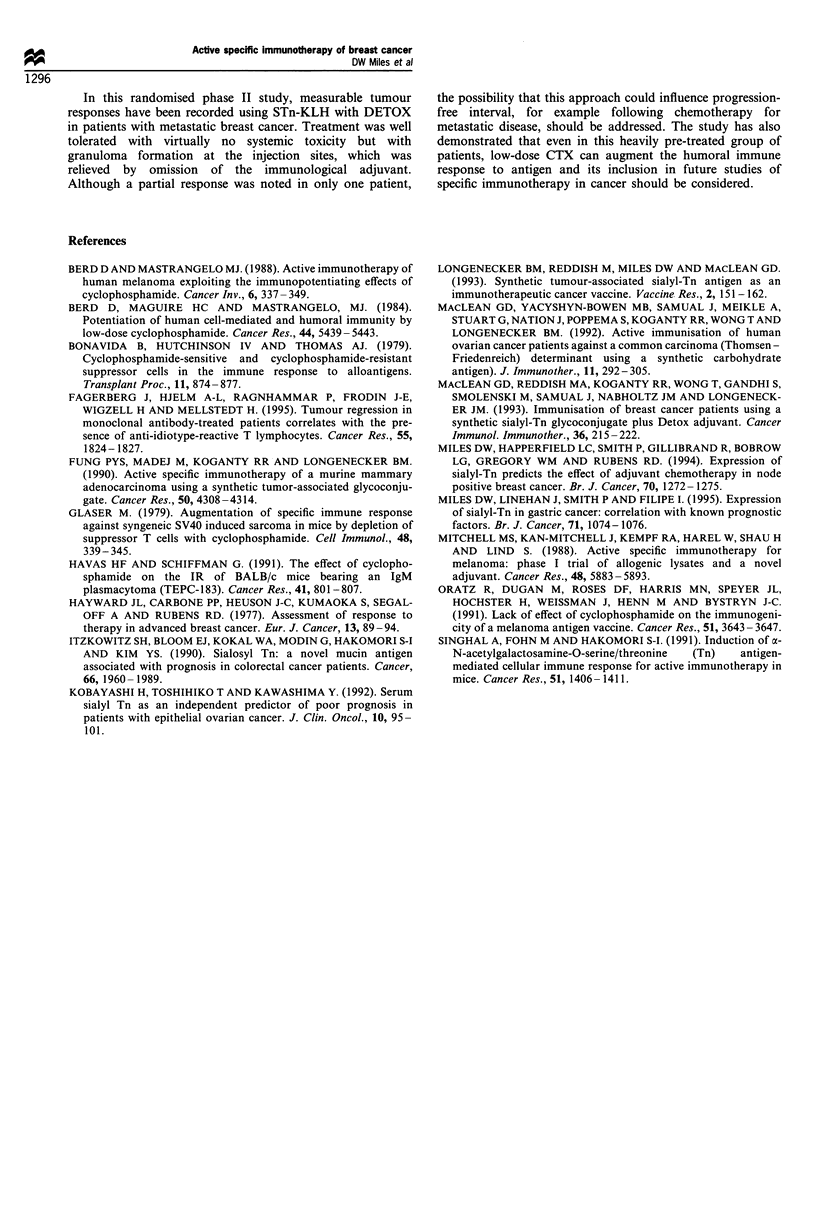# A randomised phase II study of sialyl-Tn and DETOX-B adjuvant with or without cyclophosphamide pretreatment for the active specific immunotherapy of breast cancer.

**DOI:** 10.1038/bjc.1996.532

**Published:** 1996-10

**Authors:** D. W. Miles, K. E. Towlson, R. Graham, M. Reddish, B. M. Longenecker, J. Taylor-Papadimitriou, R. D. Rubens

**Affiliations:** ICRF Clinical Oncology Unit, Guy's Hospital, London.

## Abstract

Studies in animal models of mouse mammary carcinoma have shown that ovine submaxillary mucin, which carries multiple sialyl-Tn (STn) epitopes, is effective in stimulating an immune response and inhibiting tumour growth. In similar studies using carbohydrate antigens, pretreatment with low-dose cyclophosphamide has been shown to be important in modulating the immune response to antigen possibly by inhibiting suppresser T-cell activity. In a clinical trial assessing the efficacy and toxicity of synthetic STn, patients with metastatic breast cancer were randomised to receive 100 micrograms STn linked to keyhole limpet haemocyanin (KLH) with DETOX-B adjuvant given by subcutaneous injection at weeks 0, 2, 5 and 9 with or without low-dose cyclophosphamide (CTX, 300 mg m-2) pretreatment, 3 days before the start of immunotherapy. Patients with responding or stable disease after the first four injections were eligible to receive STn-KLH at 4 week intervals. The main toxicity noted was the development of subcutaneous granulomata at injection sites. Of 23 patients randomised, 18 received four injections, 5 patients having developed progressive disease during the initial 12 week period. Two minor responses were noted in the 18 patients who received four active specific immunotherapy (ASI) injections and a further five patients had stable disease. Six patients continued ASI at 4 week intervals and a partial response was noted in a patient who had previously had stable disease. All patients developed IgG and IgM responses to sialyl-Tn and levels of IgM antibodies were significantly higher in those patients who were pretreated with CTX. Measurable tumour responses have been recorded following ASI with STn-KLH plus DETOX and the immunomodulatory properties of low-dose CTX have been confirmed.


British Journal of Cancer (1996) 74, 1292-1296
© 1996 Stockton Press All rights reserved 0007-0920/96 $12.00

### A randomised phase II study of sialyl-Tn and DETOX-B adjuvant with or without cyclophosphamide pretreatment for the active specific immunotherapy of breast cancer

DW Miles[1], KE Towlson[1], R Graham[2], M Reddish[3], BM Longenecker[3], J Taylor-Papadimitriou[2] and RD Rubens[1]

[1]*ICRF Clinical Oncology Unit, Guy's Hospital, London SE1 9RT;* [2]*Epithelial Cell Biology Laboratory, ICRF, 44 Lincoln's Inn Fields, London WC2 3PX;* [3]*Biomira Inc., Edmonton, Alberta, Canada.*

**Summary** Studies in animal models of mouse mammary carcinoma have shown that ovine submaxillary mucin, which carries multiple sialyl-Tn (STn) epitopes, is effective in stimulating an immune response and inhibiting tumour growth. In similar studies using carbohydrate antigens, pretreatment with low-dose cyclophosphamide has been shown to be important in modulating the immune response to antigen possibly by inhibiting suppresser T-cell activity. In a clinical trial assessing the efficacy and toxicity of synthetic STn, patients with metastatic breast cancer were randomised to receive 100 $\mu$g STn linked to keyhole limpet haemocyanin (KLH) with DETOX-B adjuvant given by subcutaneous injection at weeks 0, 2, 5 and 9 with or without low-dose cyclophosphamide (CTX, 300 mg m$^{-2}$) pretreatment, 3 days before the start of immunotherapy. Patients with responding or stable disease after the first four injections were eligible to receive STn-KLH at 4 week intervals. The main toxicity noted was the development of subcutaneous granulomata at injection sites. Of 23 patients randomised, 18 received four injections, 5 patients having developed progressive disease during the initial 12 week period. Two minor responses were noted in the 18 patients who received four active specific immunotherapy (ASI) injections and a further five patients had stable disease. Six patients continued ASI at 4 week intervals and a partial response was noted in a patient who had previously had stable disease. All patients developed IgG and IgM responses to sialyl-Tn and levels of IgM antibodies were significantly higher in those patients who were pretreated with CTX. Measurable tumour responses have been recorded following ASI with STn-KLH plus DETOX and the immunomodulatory properties of low-dose CTX have been confirmed.

**Keywords:** active specific immunotherapy; sialyl-Tn; breast

Sialyl-Tn (STn) is defined by the structure NANA$\alpha$(2-6)GalNAc and is a carcinoma-associated core region carbohydrate antigen of epithelial mucin. Expression of sialyl-Tn is associated with a poor prognosis in colon (Itzkowitz *et al.*, 1990), gastric (Miles *et al.*, 1995), ovarian (Kobayashi *et al.*, 1992) and breast cancer (Miles *et al.*, 1994), and may therefore be a relevant target for the potential immunotherapy of such tumours (Longenecker *et al.*, 1993).

Studies in animal models have demonstrated slowing of tumour growth and prolongation of survival following immunisation with carbohydrate antigens (Fung *et al.*, 1990; Singhal *et al.*, 1991). In a phase I study in patients with metastatic breast cancer, immunisation with a synthetic STn linked to keyhole limpet haemocyanin (KLH) and given with an immunological adjuvant (DETOX-B) led to development of hapten-specific IgM and IgG antibodies in all patients (MacLean *et al.*, 1993). In this study, 2 of 13 evaluable patients had a partial response to immunotherapy.

A potential method of increasing the immunogenicity of tumour vaccines is the use of cyclophosphamide (CTX) before immunisation. Low-dose CTX (200-300 mg m$^{-2}$) given 2-4 days before antigen administration enhances humoral and cellular responses to vaccine immunisation in tumour-bearing animals (Glaser, 1979; Havas and Schiffmann, 1981) and in man (Berd *et al.*, 1984; Fagerberg *et al.*, 1995). It has been suggested that this effect is a result of inactivation of suppressor T-lymphocytes which down-regulate responses to novel antigens (Bonavida *et al.*, 1979; Berd and Mastrangelo, 1988). In malignant melanoma, CTX pretreatment has been reported to augment cellular immunity induced by a melanoma vaccine in patients with metastatic disease (Berd and Mastrangelo, 1988) but not in patients with a low tumour burden (Oratz *et al.*, 1991).

The randomised phase II study reported here was designed to assess whether pretreatment with low-dose CTX enhanced the immune response to sialyl-Tn-KLH with DETOX-B in patients with metastatic breast cancer.

### Methods

### *Patient group*

Patients with metastatic breast cancer whose disease was assessable, who were ECOG status $\leqslant 2$ and had an expected survival of >6 months were eligible for this study. Patients were required to have a lymphocyte count of $\geqslant 1 \times 10^9$ l$^{-1}$ and to have been without systemic anti-tumour treatment or radiotherapy for 6 weeks before study entry. Patients on corticosteroids or other immunosuppressive drugs were not eligible to enter the study.

### *Active specific immunotherapy (ASI) formulation preparation*

Sialyl-Tn was provided as a sterile, pyrogen-free formulation by Biomira Inc. (Edmonton, Alberta, Canada). Each vial of STn-KLH contained 150 $\mu$g of STn-KLH in 0.75 ml of phophate-buffered saline. DETOX-B (RIBI ImmunoChem Research, Hamilton, MT, USA) is a sterile, pyrogen-free preparation (Mitchell *et al.*, 1988), and is formulated as a lyophilised oil droplet emulsion containing monophosphoryl lipid A and cell wall skeleton from *Mycobacterium phlei.* Immediately before injection, STn-KLH was added to the lyophilised DETOX. An aliquot of 0.5 ml of the finished dosage form (containing 100 $\mu$g STn-KLH) was withdrawn for injection. The administered dose consisted of 0.5 ml of

Correspondence: DW Miles
Received 8 February 1996; revised 2 May 1996; accepted 7 May 1996

the reconstituted emulsion of STn-KLH in DETOX-B. Half of the volume (0.25 ml) was administered by subcutaneous injection into two of three possible injection sites: the deltoid region of the contralateral arm and the upper thighs anterolaterally. Injection sites were rotated sequentially for the subsequent treatments. Patients were randomised to receive pretreatment with cyclophosphamide or not, 3 days before the first ASI injection (day −3). Patients randomised to cyclophosphamide pretreatment received a single intravenous dose of 300 mg $m^2$ with metoclopramide 20 mg i.v. On day 0, all patients received the first vaccination of STn-KLH in DETOX-B followed by three further injections at 2, 5 and 9 weeks. Patients who had not progressed at week 12 were eligible to receive four further injections at monthly intervals (weeks 13, 17, 21 and 25). Trial schema is illustrated in Figure 1. Patients who had stable or responding disease at week 29 could receive further injections at 3 monthly intervals until disease progression unless the patient requested withdrawal from the study.

### *Pretreatment assessment*

Prestudy investigations included full blood count and biochemical screen, chest radiograph, isotope bone scan with plain films of areas of increased tracer uptake, and liver ultrasound.

**Figure 1** Trial schema of randomised phase II study of ASI with and without cyclophosphamide (CTX) pretreatment. ASI consisted of 100 $\mu$g of STn-KLH given with DETOX-B by subcutaneous injection.

### *Response assessment*

Superficial lesions were measured clinically with calipers. Other tumour dimensions were determined by appropriate radiological investigations. Assessments were made before treatment, at week 12 and subsequently at monthly intervals according to a modification of the UICC criteria (Hayward *et al.*, 1977). Tumour responses were classified as follows: (1) complete response, disappearance of all evidence of disease; (2) partial response, ⩾50% reduction in the sum of the product of the two largest perpendicular diameters of all measurable lesions with no new lesions appearing; (3) minor response, ⩾25% but <50% reduction in tumour lesions; (4) stable disease, changes insufficient to enable classification into categories 3 or 4, i.e. a decrease in the area of all tumours less than 25% or an increase in tumour lesions less than 25%; (5) progressive disease, appearance of new lesions or a ⩾25% increase in the product of the two largest perpendicular diameters of any individual lesion.

Time to disease progression was measured from the date the patients started treatment to the date of detection of progression of previously described lesions at baseline or detection of new lesions.

### *Toxicity assessment*

Toxicity was assessed at each clinic visit and graded according to the National Cancer Institute common toxicity criteria.

### *Immune response*

Titres of anti-STn IgG and IgM antibodies were measured by quantitative enzyme-linked immunosorbent assay (ELISA) in serum samples obtained at baseline and before each vaccination. Microtitre 96-well plates were coated with STn-human serum albumin (HSA) conjugate and control plates were coated with HSA alone (values for HSA were subtracted from STn-HSA to yield the results for STn alone), ovine submaxillary mucin (OSM, a natural source of STn) and KLH. Serial dilutions of serum were incubated with antigen-coated plates at room temperature for 1 h and washed. Alkaline phosphatase-labelled specific anti-human IgG or IgM was added as second antibody. After washing, substrate *p*-nitrophenyl phosphate substrate was added to each well. The enzyme reaction was stopped with 1 M hydrochloric acid and the optical densities of the resulting solutions were measured with an automated spectrophotometer. The results of the titration are reported as endpoint dilutions of serum which reached 4.0 mO.D. $min^{-1}$.

### Results

Twenty-three patients with metastatic breast cancer were entered into this randomised phase II study of STn-KLH in DETOX-B adjuvant with or without cyclophosphamide pretreatment. Patient characteristics are illustrated in Table I. Median age of the patient group was 56 years (range 36–73 years) and initial disease-free interval was 42 months (range 9–89 months). Seventeen patients had received prior endocrine therapy and 12 patients prior chemotherapy for metastatic disease. Twenty patients had ⩽two sites of disease and the majority of sites were nodal and cutaneous deposits. There were no significant differences in the above parameters between the two patient groups (Mann–Whitney *U* and Fisher's exact tests).

Eighteen patients completed at least four vaccinations according to the protocol. Five patients were withdrawn from the study before the first formal assessment because of progressive disease which required alternative systemic therapy.

Of the 18 patients assessed at week 12, 11 had progressive disease and five had stable disease. Two patients achieved a minor response (<50% but >25% reduction in tumour size). Both these patients were in the no cyclophosphamide pretreatment group and had small bulk metastatic disease. Patient 003, whose prior systemic treatment with tamoxifen had been discontinued 8 months before commencing ASI, had a minor response in a single supraclavicular fossa node which lasted 3 months. Following the second monthly injection, progressive disease in the lymph node was noted and coincided with appearance of bony metastases. Patient 022, who had progressive disease following tamoxifen, had a minor response in a single pulmonary nodule, which is maintained 15 months following the start of ASI. Following the four monthly injections, this patient continues to recieve ASI at 3 monthly intervals.

Six patients received further ASI at monthly intervals and were assessed for response at week 29. One patient, in the cyclophosphamide pretreatment group whose disease was stable at the week 12 assessment, achieved a partial response of 3 months' duration in breast and cutaneous disease. One other patient had stable disease for a period of 8 months and the other four patients had progressive disease.

### *Toxicity*

Toxicity associated with cyclophosphamide administration was transient mild to moderate nausea in all patients and transient vomiting (CTC grade II) in two patients.

Following injection of STn-KLH plus DETOX-B, all patients developed erythema at injection sites which peaked at 24–48 h after injection and resolved completely after a few days. Seventeen patients developed granulomata at the injection sites which increased in size with subsequent injections of ASI (maximum median diameter 10 mm, range 5–60 mm, no significant differences between the two groups, Mann–Whitney *U*-test). In nine patients ulceration of the granulomata occurred and in four patients DETOX-B was omitted from subsequent injections, during the period of monthly injections. This resulted in less granuloma formation and no ulceration.

Systemic toxicity was limited to mild 'flu-like symptoms in two patients in the immediate post-treated period (24–72 h) which were treated symptomatically with paracetamol.

**Table I** Patient characteristics

| | Treatment group *Group A* *(CTX preRx)* | *Group B* *(no CTX preRx)* |
|---|---|---|
| Age (years) | | |
| Median | 59 | 54 |
| Range | 36–68 | 43–73 |
| Disease-free interval (months) | | |
| Median | 40 | 44 |
| Range | 9–90 | 11–79 |
| Sites of diseases | | |
| Lymph nodes | 7 | 6 |
| Skin | 8 | 3 |
| Lung | 2 | 6 |
| Bone | 3 | 1 |
| Pleura | 2 | 0 |
| Breast | 2 | 0 |
| Liver | 0 | 1 |
| Ovary | 1 | 1 |
| Number of disease sites | | |
| 1 | 2 | 5 |
| 2 | 8 | 5 |
| 3 | 2 | 1 |
| Prior hormone therapy (number of lines of Rx) | | |
| 0 | 3 | 1 |
| 1 | 2 | 6 |
| 2 | 5 | 2 |
| 3 | 2 | 2 |
| Prior chemotherapy (number of lines Rx) | | |
| 0 | 4 | 7 |
| 1 | 5 | 0 |
| 2 | 3 | 2 |
| 3 | 0 | 2 |

### *Antibody responses*

The development of serum IgM and IgG antibodies to STn (conjugated to human serum albumin, HSA), ovine submaxillary mucin ( which bears multiple STn epitopes) and to KLH were measured by quantitative ELISA before ASI, at each treatment course and at the formal assessment of tumour response (week 12). All patients developed IgG and IgM responses to sialyl-Tn antigens, although levels of anti-STn IgG antibodies were higher when measured with STn-HSA compared with OSM. High levels of IgG antibodies to the glycoprotein carrier KLH were noted in all patients. No significant differences between STn or KLH antibody levels were noted according to whether or not the patients had received prior chemotherapy for metastatic disease. Although levels of IgG antibodies appeared to be higher from week 5 onwards in the cyclophosphamide pretreatment group (Figure 2), the differences were not statistically significant. The levels of IgM antibodies to STn-HSA and OSM were significantly higher in the group which had received cyclophosphamide pretreatment (Figure 2).

### Discussion

Specific humoral immune responses to carbohydrate antigens have been demonstrated previously in animal models and patients with breast, ovarian and colorectal cancer. Studies using synthetic carbohydrate antigens in patients with breast and ovarian cancer have previously demonstrated the specificity of the anti-hapten humoral immune response (MacLean *et al.*, 1992, 1993). In the phase I study of sialyl-Tn in patients with metastatic breast cancer, two partial responses were noted in 13 patients and two patients had mixed responses (reduction in tumour burden at some sites and progression in other sites; MacLean *et al.*, 1993).

Studies in animal models have suggested that the immune response may be augmented by pretreatment with low-dose cyclophosphamide, possibly by inhibition of a subset of putative suppressor T-lymphocytes (Mastrangelo and Berd, 1988). Studies comparing the effects of i.v. CTX *vs* no pretreatment on cell-mediated immunity in patients receiving vaccine therapy for melanoma have yielded contradictory results (Mastrangelo and Berd, 1988; Oratz *et al.*, 1991) possibly as a result of differences in tumour burden at the time of treatment.

We report here the results of a randomised phase II study assessing the effect of low-dose cyclophosphamide pretreatment on the immune response to the synthetic carbohydrate antigen, sialyl-Tn, conjugated to KLH and given with an immunological adjuvant. The systemic toxicity of the treatment schedule was low with only transient nausea in those receiving CTX and mild 'flu-like symptoms associated with ASI injections. The main toxicity of ASI was the development of granulomas at injection sites and subsequent ulceration. Omission of DETOX-B from the ASI formulation alleviated this problem during subsequent treatment courses. Measurable reductions in tumour burden were noted in two patients following the four initial ASI injections, although changes in dimensions of measurable lesions were not sufficient to be categorised as a partial response according to standard UICC criteria. Both these minor responses occurred in patients who did not receive CTX pretreatment and who had minimal bulk metastatic disease. A partial response was noted in a breast mass following four further ASI injections and, although this occurred in a patient who received CTX pretreatment, the response was documented 29

weeks following the CTX pretreatment and could not therefore be attributed to a direct anti-tumour action of this drug.

Following STn-KLH with DETOX, all patients developed IgM and IgG responses to sialyl-Tn, although antibody responses were lower when measured using OSM compared with synthetic hapten. This has been noted previously (Longenecker *et al.*, 1993) and is probably caused by differences in antibody affinity. Patients also developed antibody responses to the glycoprotein carrier, KLH. Although half the patients in the group had received at least one line of chemotherapy for metastatic disease, its use did not influence immune responsiveness in terms of antibody tires to STn or KLH. Low-dose cyclophosphamide (300 mg $m^{-2}$) did, however, influence the humoral immune response. Induced levels of IgG to STn and KLH appeared to be higher in the CTX pretreatment group, although the apparent differences were not statistically significant. Levels of IgM antibodies to STn were, however, significantly higher in the CTX pretreatment group. Although levels of IgM antibodies to KLH appeared to be higher in the CTX pretreatment group, the differences were not statistically significant. There was no obvious correlation between induced levels of antibodies to STn and clinical response, although comparison is necessarily difficult in view of the small numbers involved.

**Figure 2** Antibody responses (IgG and IgM) to sialyl-Tn conjugated to human serum albumin (STn-HSA), ovine submaxillary mucin (OSM) and keyhole limpet haemocyanin (KLH) in patients receiving active specific immunotherapy (ASI) with STn-KLH plus DETOX, with (□) and without (◆) cyclophosphamide (CTX) pretreatment (300 mg $m^{-2}$, day −3). ASI injection schedule is represented by arrows. Levels of IgM antibodies to STn-HSA and OSM were significantly higher in the CTX pretreated group (*$P<0.01$, **$P<0.02$, $^{+}P<0.05$, Mann–Whitney $U$-test).

In this randomised phase II study, measurable tumour responses have been recorded using STn-KLH with DETOX in patients with metastatic breast cancer. Treatment was well tolerated with virtually no systemic toxicity but with granuloma formation at the injection sites, which was relieved by omission of the immunological adjuvant. Although a partial response was noted in only one patient, the possibility that this approach could influence progression-free interval, for example following chemotherapy for metastatic disease, should be addressed. The study has also demonstrated that even in this heavily pre-treated group of patients, low-dose CTX can augment the humoral immune response to antigen and its inclusion in future studies of specific immunotherapy in cancer should be considered.

### References

BERD D AND MASTRANGELO MJ. (1988). Active immunotherapy of human melanoma exploiting the immunopotentiating effects of cyclophosphamide. *Cancer Inv.*, **6**, 337–349.

BERD D, MAGUIRE HC AND MASTRANGELO, MJ. (1984). Potentiation of human cell-mediated and humoral immunity by low-dose cyclophosphamide. *Cancer Res.*, **44**, 5439–5443.

BONAVIDA B, HUTCHINSON IV AND THOMAS AJ. (1979). Cyclophosphamide-sensitive and cyclophosphamide-resistant suppressor cells in the immune response to alloantigens. *Transplant Proc.*, **11**, 874–877.

FAGERBERG J, HJELM A-L, RAGNHAMMAR P, FRODIN J-E, WIGZELL H AND MELLSTEDT H. (1995). Tumour regression in monoclonal antibody-treated patients correlates with the presence of anti-idiotype-reactive T lymphocytes. *Cancer Res.*, **55**, 1824–1827.

FUNG PYS, MADEJ M, KOGANTY RR AND LONGENECKER BM. (1990). Active specific immunotherapy of a murine mammary adenocarcinoma using a synthetic tumor-associated glycoconjugate. *Cancer Res.*, **50**, 4308–4314.

GLASER M. (1979). Augmentation of specific immune response against syngeneic SV40 induced sarcoma in mice by depletion of suppressor T cells with cyclophosphamide. *Cell Immunol.*, **48**, 339–345.

HAVAS HF AND SCHIFFMAN G. (1991). The effect of cyclophosphamide on the IR of BALB/c mice bearing an IgM plasmacytoma (TEPC-183). *Cancer Res.*, **41**, 801–807.

HAYWARD JL, CARBONE PP, HEUSON J-C, KUMAOKA S, SEGALOFF A AND RUBENS RD. (1977). Assessment of response to therapy in advanced breast cancer. *Eur. J. Cancer*, **13**, 89–94.

ITZKOWITZ SH, BLOOM EJ, KOKAL WA, MODIN G, HAKOMORI S-I AND KIM YS. (1990). Sialosyl Tn: a novel mucin antigen associated with prognosis in colorectal cancer patients. *Cancer*, **66**, 1960–1989.

KOBAYASHI H, TOSHIHIKO T AND KAWASHIMA Y. (1992). Serum sialyl Tn as an independent predictor of poor prognosis in patients with epithelial ovarian cancer. *J. Clin. Oncol.*, **10**, 95–101.

LONGENECKER BM, REDDISH M, MILES DW AND MACLEAN GD. (1993). Synthetic tumour-associated sialyl-Tn antigen as an immunotherapeutic cancer vaccine. *Vaccine Res.*, **2**, 151–162.

MACLEAN GD, YACYSHYN-BOWEN MB, SAMUAL J, MEIKLE A, STUART G, NATION J, POPPEMA S, KOGANTY RR, WONG T AND LONGENECKER BM. (1992). Active immunisation of human ovarian cancer patients against a common carcinoma (Thomsen–Friedenreich) determinant using a synthetic carbohydrate antigen). *J. Immunother.*, **11**, 292–305.

MACLEAN GD, REDDISH MA, KOGANTY RR, WONG T, GANDHI S, SMOLENSKI M, SAMUAL J, NABHOLTZ JM AND LONGENECKER JM. (1993). Immunisation of breast cancer patients using a synthetic sialyl-Tn glycoconjugate plus Detox adjuvant. *Cancer Immunol. Immunother.*, **36**, 215–222.

MILES DW, HAPPERFIELD LC, SMITH P, GILLIBRAND R, BOBROW LG, GREGORY WM AND RUBENS RD. (1994). Expression of sialyl-Tn predicts the effect of adjuvant chemotherapy in node positive breast cancer. *Br. J. Cancer*, **70**, 1272–1275.

MILES DW, LINEHAN J, SMITH P AND FILIPE I. (1995). Expression of sialyl-Tn in gastric cancer: correlation with known prognostic factors. *Br. J. Cancer*, **71**, 1074–1076.

MITCHELL MS, KAN-MITCHELL J, KEMPF RA, HAREL W, SHAU H AND LIND S. (1988). Active specific immunotherapy for melanoma: phase I trial of allogenic lysates and a novel adjuvant. *Cancer Res.*, **48**, 5883–5893.

ORATZ R, DUGAN M, ROSES DF, HARRIS MN, SPEYER JL, HOCHSTER H, WEISSMAN J, HENN M AND BYSTRYN J-C. (1991). Lack of effect of cyclophosphamide on the immunogenicity of a melanoma antigen vaccine. *Cancer Res.*, **51**, 3643–3647.

SINGHAL A, FOHN M AND HAKOMORI S-I. (1991). Induction of α-N-acetylgalactosamine-O-serine/threonine (Tn) antigen-mediated cellular immune response for active immunotherapy in mice. *Cancer Res.*, **51**, 1406–1411.